# A Randomized Controlled Trial of Two Schedules of Hepatitis B Vaccination in Predialysed Chronic Renal Failure Patients

**DOI:** 10.5812/hepatmon.6438

**Published:** 2012-05-30

**Authors:** Farokhlagha Ahmadi, Morteza Ramezani, Effat Razeghi, Neda Ranjbarnovin, Zahra Khazaeipour

**Affiliations:** 1 Nephrology Research Center, Imam Khomeini Hospital Complex, Tehran University of Medical Sciences, Tehran, IR Iran; 2 Department of Nephrology, Sina Hospital, Tehran University of Medical Sciences, Tehran, IR Iran; 3 Brain and Spinal Injury Research Center, Imam Khomeini Hospital Complex, Tehran University of Medical Sciences, Tehran, IR Iran

**Keywords:** Kidney Failure, Chronic, Hepatitis B, Vaccination

## Abstract

**Background:**

Patients with chronic renal disease should be vaccinated as soon as dialysis is forestalled, and this could improve the seroconversion of hepatitis B vaccination.

**Objectives:**

In this study, we aimed to compare seroconversion and immune response rates using 4 doses of 40 μg and 3 doses of 20 μg Euvax B recombinant Hepatitis B surface Antigen (HBs Ag) vaccine administered to predialysis patients with chronic kidney disease (CKD).

**Patients and Methods:**

In an open, randomized clinical trial, we compared seroconversion rates in 51 predialysis patients with mild and moderate chronic renal failure who received either 4 doses of 40 μg or 3 doses of 20 μg of Euvax B recombinant hepatitis B vaccine administered at 0, 1, 2, 6 and 0, 1, 6 months, respectively.

**Results:**

Differences in seroconversion rates after 4 doses of 40 μg (80.88%) compared to 3 doses of 20 μg (92%) were not significant (P = 0.4124). The mean HBs antibody level after 4 doses of 40 μg at 0, 1, 2, and 6 months (182.2 ± 286.7) was significantly higher than that after 3 doses of 40 μg at 0,1, and 6 months (96.9 ± 192.1) (P = 0.004). Seroconversion after 4 doses of 40 μg (80.8%) was also significantly higher than that after 3 doses of 40 μg (77%) (P = 0.004). Multivariable analysis showed that none of the variables contributed to seroconversion.

**Conclusions:**

We found that 4 doses of 40 μg did not lead to significantly more seroconversion than 3 doses of 20 μg.

## 1. Background

Chronic kidney disease (CKD) and its treatment enhance the risk of Hepatitis B Virus (HBV) infection [1]. Since 1982, the HBV vaccine has been recommended for CKD patients and healthcare workers [2][3][4][5]. Patients with CKD are immunocompromised, whether they are on dialysis or not, and have lower immune response rates (50–80%) to the vaccine when compared with immunocompetent individuals (95%) [6][7][8]. Different strategies have been employed to improve the immune response in CKD patients, such as increasing the number of intramuscular (i.m.) doses [9], increasing the quantity of antigen in each dose [10], intradermal administration [11], and co-administration of immunogenic or immunostimulant agents such as zinc [12], levamizole [13], gammainterferon [14] interleukin-2 [15], granulocyte colony stimulating factor, and erythropoietin [16]. To improve vaccination response, it is advised that CKD patients should be vaccinated as early in the course of renal disease as possible [17] using a double vaccine dose [17][18] and a 4- rather than 3-dose schedule [6]. Several studies in predialysis patients that have compared vaccine responses to the different doses have been inconclusive [19][20][21]. The current vaccination strategy for predialysis patients at our centers (Imam Khomeini hospital complex and Sina hospital) is 20 μg (1 ml) administered at 0, 1, and 6 months. We aimed to test a new vaccination schedule because we thought the current method ineffective. In this study, we aimed to compare seroconversion rates (Hepatitis B surface Antibody [HBs Ab] ≥ 10 mIU/ml) using 4 doses of 40 μg and 3 doses of 20 μg of Euvax B recombinant Hepatitis B surface Antigen (HBs Ag) vaccine administered to predialysis CKD patients.

## 2. Patients and Methods

### 2.1. Sample Size

In accordance with the McNulty et al. study [22], to perform an analysis with 80% power using a two-sided alpha level (α = 0.05) and assuming 66% and 81% seroconversion with the 20 μg and 40 μg hepatitis B vaccine, we needed to enroll 137 patients. All the available patients who met the inclusion criteria were recruited for this study.

### 2.2. Patients

The patients were recruited for the study from the renal outpatient clinics (Imam Khomeini hospital complex and Sina hospital) at Tehran University of Medical Sciences, which provide diagnostic and follow-up services for patients with renal disease, between October 2008 and October 2009. Inclusion criteria were age > 18 years, mild or moderate chronic renal failure (a serum creatinine level = 1.5–6 mg/dl). Exclusion criteria were severe renal failure (a serum creatinine level > 6 mg/dl) requiring dialysis or expected to require dialysis within 1 year, patients with positive HBs Ag, HBs Ab, or HBc Ab, patients receiving immunosuppressive treatment, and those with known lymphoproliferative disorder.

### 2.3. Trial Protocol

Records were made of demographic details, drug therapy, and the weight and height of the patients. Baseline blood samples were taken to determine the full blood count and blood chemistry, including Fasting Blood Sugar (FBS), C Reactive Protein (CRP), intact Parathyroid Hormone (iPTH), lipid profile, Albumin (Alb), Creatinin (Cr), Ferritin, and hepatitis B and C markers. Randomization process: A 4-block randomized method was used. Patients were randomized to receive 3 doses of 20 μg (1 ml) or 4 doses of 40 μg (2 ml) of Euvax B vaccine (recombinant hepatitis B surface antigen adsorbed on aluminum hydroxide adjuvant; LG Life Sciences). All cases were followed until the vaccination processes were completed, and there were no withdrawals. Blinding: Blinding was not possible, because the frequency of vaccination in the two groups was unequal. The vaccine was administered (i.m.) into the deltoid muscle using a 23G × 1” needle at 0, 1, and 6 months (3-dose group) and at 0, 1, 2, and 6 months (4-dose group). Patients were followed to assess routine laboratory markers of chronic kidney disease (Glomerular filtration rate [GFR], Cr, Na, K, Ca, P, and so on) and a clinical examination was performed every 3 months. GFR was calculated using the Cockcroft-Gault formula, where GFR ml/min = (140-age) × weight × K / Cr × 72 and K = 1 (for males) or 0.85 (for females). Blood samples were taken before 6 months as well as 8–10 weeks after the 6-month dose, and were assayed for the presence of antibodies against HBs Ag (anti-HBs) using a commercial enzyme immunoassay (EIA) produced by Abbott Laboratories (AUSAB, Abbott Laboratories, Abbott Park, IL, USA).

### 2.4. Hepatitis B Markers

Hepatitis B surface antibody was determined using an anti-hepatitis B surface antibody quantitative chemiluminescence assay (EIA) produced by Abbott Laboratories (AUSAB, Abbott Laboratories, Abbott Park, IL, USA) at Noor laboratory, Tehran, Iran. Anti-HBs titers are expressed in mIU/ml compared with standard immunoglobulin preparations (Ortho- Clinical Diagnostics). Anti-HBs titers < 10 mIU/ml were defined as non-seroconversion (non-responder). Anti-HBs titers ≥ 10 mIU/ml were defined as seroconversion.

### 2.5. Data Analysis

After evaluating the normality of the data using a One-Sample Kolmogorov-Smirnov Test, quantitative variables for the 2 qualitative groups were analyzed using a Mann-Whitney U test. The Wilcoxon Signed Ranks test was used for before and after comparisons of quantitative data. Associations between qualitative data were tested using a Chi-square test. Correlations between 2 quantitative variables were evaluated by Spearman’s correlation analysis. Logistic regression analysis model was used to evaluate predictors of response to vaccination. In this model , responses to 3 doses of 20 μg and 4 doses of 40 μg of HBs Ab (< 10 and ≥ 10 mIU/ml) were dependent variables and type of vaccination, age, ferritin, FBS, HDL, Alb, weight, and HCV Ab were independent variables. Quantitative variables were presented as mean ± standard deviation (SD). The significance level was set at P ≤ 0.005. Ethics: Since 1996, the English National Immunization Guide [8] has recommended that “in addition to those already on hemodialysis, the immunization of all patients with chronic renal failure is recommended, as soon as it is anticipated they may require dialysis”. An information sheet explaining the randomization process, the vaccination dose, follow-up, and the rationale for the study was provided to the patients. We obtained their written consent to participate in the study after they had read the information leaflet and discussed it with us. The study was approved by the research ethics committee of Tehran University of Medical Sciences.

## 3. Results

Of 64 predialysis patients, 51 completed the 8-month follow-up (26 patients received 4 doses of 40 μg, and 25 patients 3 doses of 20 μg). Two of the remaining patients died, 2 required dialysis, and 9 were eliminated from the study because of problems reaching Tehran from other cities. There were no significant differences in the frequency and etiology of withdrawal between the 2 groups. The patients all had stage 3 or 4 renal failure, but gender differed significantly between the 2 groups: 8 patients (32.0%) in the 3-dose group and 18 (69.2%) in the 4-dose group were male (P = 0.008). Analysis of the age, weight, serum Cr, Alb, Hb, CRP, PTH, ferritin, FBS, LDL, HDL, cholesterol, and GFR of the 51 patients at enrolment did not reveal any significant differences between the 2 study groups (Table 1). The etiology of renal disease was as follows: glomerulonephritis 9.8%, diabetes mellitus 13.7%, polycystic kidney disease 11.7%, hypertension 35.3%, diabetes mellitus and hypertension 9.8%, benign prostatic hypertrophy 9.9%, and unknown etiology 9.8%. There were no significant differences in the etiology of renal disease between the 2 groups. Three patients in the 3-dose group (12%) and 4 in the 4-dose group (15.4%) had diabetes mellitus (P = 0.52). In addition, 3 patients in the 3-dose group were HCV Ab positive.

**Table 1:** Description of Demographic and Clinicopathologic Characteristics of Study Groups

	**20 µg - 3 Dose Group (n = 25)**	**40 µg - 4 Dose Group (n = 26)**	***P***** value **b
Gender, No.			0.008 c
Male	8	18	
Female	17	8	
Weight, kg, mean ± SD	72.6 ± 9.3	69.7 ± 6.6	0.12
Age, y, mean ± SD	56.9 ± 13.0	57.1 ± 11.9	0.76
GFR a, ml/min, mean ± SD	31.8 ± 10.9	34.9 ± 13.9	0.46
Albumin, g/dl, mean ± SD	4.6 ± 0.4	4.4 ± 0.4	0.10
Creatinine, mg/dl, mean ± SD	2.7 ± 1.0	2.6 ± 0.8	0.95
Hb a, g/dl, mean ± SD	11.6 ± 2.0	12.3 ± 1.9	0.52
CRP a, mg/dl, mean ± SD	2.9 ± 4.4	3.4 ± 4.5	0.84
PTH a, pmol/l, mean ± SD	153.2 ± 149.0	103.9 ± 78.2	0.27
Ferritin, ng/ml, mean ± SD	120.0 ± 94.6	132.8 ± 113.6	0.77
FBS a, mg/dl, mean ± SD	105.9 ± 34.2	111.2 ± 34.9	0.22
LDL a, mg/dl, mean ± SD	100.8 ± 30.9	87.4 ± 24.9	0.10
HDL a, mg/dl, mean ± SD	42.0 ± 12.8	44.9 ± 16.3	0.59
Cholesterol, mg/dl, mean ± SD	185.7 ± 44.2	170.8 ± 39.6	0.19

^a^ Abbreviations: CRP, C Reactive Protein; FBS, Fasting Blood Sugar; GFR, Glomerular filtration rate Hb, Hepatitis B; HDL, High-density lipoprotein; LDL, Low-density lipoproteinm, PTH, Parathyroid Hormone; GFR, Glomerular filtration rate

^b^ Mann-Whitney U Test

^c^ Chi- Square Test

The mean HBs Ab level after 4 doses of the 40 μg vaccine (182.2 ± 286.7) was higher than that after 3 doses of the 20 μg vaccine (107.6 ± 192.1), but the difference was not significant (P = 0.3). After 4 doses of the 40 μg vaccine, the cumulative seroconversion rate (HBs Ab ≥ 10 mIU/ml) was not significantly different (21/26, 80.8%) than that after 3 doses of 20 μg (23/25, 92%) P = 0.41). The mean HBs Ab level after 4 doses of 40 μg (182.2 ± 286.7) was significantly higher than that attained after 3 doses of the 40 μg vaccine (96.9 ± 192.1) (P = 0.004). In addition, after 4 doses of 40 μg (21/26, 80.8%), the seroconversion rate was higher than that after 3 doses of 40 μg (20/26, 77%). There was no correlation between age and serum HBs Ab level in the 3-dose group; however, there was a negative correlation in the 4-dose group (r = -0.58, P = 0.002).

### 3.1. Multivariable Analysis

In the logistic regression analysis (Table 2), there was no difference between the group receiving 3 doses of 20 μg and those receiving 4 doses of 40 μg of HBV vaccine in terms of predicting the proportion with seroconversion, adjusting for age, ferritin, FBS, HDL, Alb, weight, and HCV Ab.

**Table 2:** Logistic Regression Analysis to Determine Predictors of Antibody Response (HBs Ab ≥ 10 mIU/ml) After 3 Doses of 20 µg and 4 Doses of 40 µg, HBV Vaccination

	***P *value**	**OR a**	**95.0% CI For OR**	
			**Lower**	**Upper**
2 vaccination schedule	0.17	0.16	0.01	2.18
Age	0.53	1.05	0.90	1.21
Ferritin	0.18	1.03	0.99	1.07
FBS a, b	0.52	2.90	0.11	77.18
HDL^a^, ^c^	0.49	0.33	0.01	8.14
Albumin^d^	0.28	8.55	0.17	433.64
Weight	0.07	1.22	0.98	1.51
HCV Ab(+/-)	0.46	0.32	0.01	6.76
Constant	0.17	0.000		

^a^ Abbreviations: FBS, Fasting Blood Sugar; HDL, High-density lipoprotein

^b^ FBS ≤ 100 is based

^c^ HDL ≤ 40 is based

^d^ Albumin ≤ 4 is based

## 4. Discussion

Patients with renal insufficiency have a suboptimal response to HBV vaccination, and frequent boosters are needed to maintain protection [23][24]. We compared 2 HBV vaccination schedules, namely 20 μg at 0, 1, and 6 months and 40 μg at 0, 1, 2, and 6 months. There was no significant difference in the seroconversion rate between the 40 μg 4-dose vaccination group (21/26, 80.8%) and the 20 μg 3-dose group (23/25, 92%). These findings support the McNulty et al. study results [22]. In that study, 3 doses of 40 μg (67%) attained equivalent seroconversion to 3 doses of 20 μg.(57%) [22]. In our study, the mean HBs Ab level after 4 doses of 40 μg (182.2 ± 286.7) was significantly higher than that after 3 doses of 40 μg (96.9 ± 192.1) (P = 0.004). In addition, seroconversion was greater after 4 doses of 40 μg (21/26, 80.8%) compared with after 3 doses of 40 μg (20/26, 77%). As reported in some other studies [25][26], dialysis patients who did not seroconvert after the 3-dose course often did seroconvert after the fourth dose. Agarwal et al. [26] compared 2 HBV 40 μg vaccination schedules among patients with mild, moderate, and severe chronic renal failure (CRF): 0,1, and 2 months (3-dose group) vs. 0,1,2, and 6 months (4-dose group). In the 3-dose group, the seroconversion rate among those with mild, moderate, and severe CRF was 87.5%, 66.6%, and 35.7%, respectively, and in 4-dose group it was 100%, 77%, and 36.36%. They concluded that patients with CRF should be vaccinated at the very early stage of the disease using 40 μg of vaccine, and that 4 doses is better than 3 doses [26].

McNulty et al. showed that the median age of the seroconverter group was about 7.5 years lower than that of the non-seroconverter group; however, this difference was not significant [22]. Fabrizi et al. [27] conducted a meta-analysis of 17 clinical trials involving end-stage renal disease patients, and found an association between older age and impaired response to the HBV vaccine. In another study in Iran, higher age (> 60 years) was negatively correlated with seroconversion (odds ratio = 0.22; P = 0.004). [28] In our study, there was no correlation between age and serum HBs Ab level in the 3-dose group; however, there was a negative correlation in the 4-dose group (r= -0.58, P = 0.002). Similar to other reports [22][29], in this study, there was no noticeable association between GFR and seroconversion rate. The sample size was too small to notice a significant difference. A study investigating the effect of renal function on seroconversion rates suggested that CKD patients with higher GFR levels were more likely to respond to vaccination [30]. The overall vaccine response rate was 86.27%. This was similar to DaRoza et al.’s study [30]. The seroconversion rate in the Hashemi et al. study was 78%, (lower than the results of our study) [28]. Edey et al. [31] in a review article explained that the 3-dose schedule of HBV vaccine in immunocompetent individuals may lead to a 90–95% seroprotection rate; however, in patients with renal failure, the rates were lower. They suggested that dialysis patients should receive higher vaccine doses such as 40 µg at 0, 1, and 6 months, or 40 µg at 0, 1, 2, and 6 months to improve immune response. In this review, the best reported response rates to these schedules were < 85% [31]. Joukar et al. [32], in a study of hemodialysis patients in Guilan, north of Iran, concluded that despite the low rate of HBV infection in that region, HBV vaccination of end-stage renal disease patients before chronic hemodialysis can prevent HBV infection [32].

In our study, seroconversion (HBs Ab ≥ 10 mIU/ml) in the 40μg 4 dose group was 80.8%, and in 20 μg 3 dose group was 92%, (P = 0.41).

The mean antibody titers after three doses of 20 μg vaccine (107.6 ± 192.1), was lower than four doses of 40 μg (182.2 ± 286.7), but was not significant.

We hypothesized that four doses of 40μg may be better than three doses of 20μg, but we did not find any difference between two methods. These results may be related to small sample size, large standard deviation of HBs Ab levels, and lack of statistical power.

Limitations of the study: The sample size available for this time of study regarding to inclusion and exclusion criteria and time period did not reach to calculated sample size.

## 5. Conclusion

We found that the 4-dose 40 μg HBV vaccine did not lead to significantly greater seroconversion than 3 doses of 20 μg. Thus, it is perhaps not necessary to introduce a 4-dose 40 μg HBV vaccination schedule to replace the 3-dose 20 μg schedule for predialysis vaccination of patients with stage 3 and 4 CKD. However, further studies with a larger sample size are required.
